# Molecular and cellular evidence of natural Venezuelan equine encephalitis virus infection in frugivorous bats in Colombia

**DOI:** 10.14202/vetworld.2020.495-501

**Published:** 2020-03-16

**Authors:** Camilo Guzmán, Alfonso Calderón, Teresa Oviedo, Salim Mattar, José Castañeda, Virginia Rodriguez, Luiz Tadeu Moraes Figueiredo

**Affiliations:** 1 Department of Pharmacy, Faculty of Health Sciences, Institute of Biological Research of the Tropics, University of Córdoba, Colombia; 2 Faculty of Veterinary Medicine and Animal, Institute for Biological Research in the Tropics, University of Córdoba, Colombia; 3 University of Córdoba, Colombia; 4 Faculty of Veterinary Medicine and Animal, Institute of Biological Research of the Tropics, University of Córdoba, Colombia; 5 ICA Diagnostic Center - Córdoba, Colombia; 6 Faculty of Health Sciences, University of Córdoba, Colombia; 7 Center for Virological Research, University of Sao Paulo, Riberao Preto, Brazil

**Keywords:** *Alphavirus* infections, *Chiroptera*, pathology

## Abstract

**Background and Aim:**

Venezuelan equine encephalitis virus (VEEV) is an alphavirus that causes encephalitis with a high impact on public health in Latin America. However, only in Guatemala, Trinidad and Tobago, and Mexico have found antibodies in VEEV in bats, using immunohistochemistry, the sensitivity and specificity are improved; thus, it is better for demonstrating natural infection in bats as potential hosts. This study aimed to determine the presence of VEEV in tissues of frugivorous bats.

**Materials and Methods:**

A prospective descriptive cross-sectional study with a non-probabilistic sampling was carried out in 12 localities of Córdoba and Sucre area of the Colombian Caribbean. Two hundred and eighty-six bats were captured using fog nets, and the specimens according to taxonomic keys were classified. According to the Ethics Committee of the University of Córdoba, the bats were treated with analgesics and anesthetics. Blood samples were taken and then euthanized to obtain tissues and organs which were preserved in liquid N2 at −196°C. A portion of each organ was fixed in 10% buffered formalin for the detection of antigens by immunohistochemistry. Several pathological anatomy analyses were performed to determine the histological characteristics of tissue lesions of frugivorous bats naturally infected with the VEEV.

**Results:**

Of the 286 bats captured, 23 species were identified. In samples of the brain, spleen, and lung of two frugivorous bats (2/286=0.70%) *Artibeus planirostris* and *Sturnira lilium*, the presence of VEEV was confirmed by immunohistochemistry.

**Conclusion:**

A fragment of the nsP4 non-structural protein gene corresponding to the alphavirus was amplified. Two samples were positive (2/286=0.70%) in frugivorous bats; *A. planirostris* (code GenBank: MG820274) and *S. lilium* (code GenBank: MG820275). The present study showed the first molecular evidence and cellular evidence (histopathology and immunohistochemistry) of natural VEEV infection in frugivorous bats in Colombia; these bats could be a host of this zoonosis.

## Introduction

Venezuelan equine encephalitis virus (VEEV) is a positive-sense virus RNA of the family *Togaviridae* and the genus *Alphavirus*, within this same group are the equine encephalitis viruses (EEV) of the East and West, Mayaro, Madariaga, Mucambo, and Everglades [1,2]. Venezuelan equine encephalitis is an emerging infectious disease in Latin America [3,4]. There are several strains of VEEV closely related that have been classified into two epidemiological groups: Enzootic and epizootic strains [3,5]. Enzootic strains (Subtypes I, DF varieties, and Subtypes II-VI) are regularly isolated in lowland and tropical forests in Florida, Mexico, Central, and South America, where the *Culex* vector mosquito (*Melanoconiun*) spp.[3,6,7].

On the other hand, strains of the VEEV epizootic cycle (Subtype I, varieties AB and C), which are responsible for the main outbreaks in humans and equines, use several species of mosquito vectors and equines, are the highly efficient amplification hosts [8]. The outbreaks have been registered for decades in countries with enzootic circulation. The implementation of surveillance systems has allowed the detection of additional human cases in countries and areas with previously unknown VEEV activity. The enzootic subtypes of VEEV are frequently detected and isolated in habitats where they circulate in rodent reservoirs and mosquitoes. The main reservoirs are rodents of wild species of *Oryzomys*, *Zigodontomys*, *Heteromys*, *Peromyscus*, and *Proechimys*. These animals become infected and develop viremia that is sufficient to infect the vectors [9,10]. Different studies have reported evidence of VEEV infection, suggesting that bats (frugivorous, sanguinivorous, and insectivorous) could be host to arboviruses of public health impact in America [11-15]. Clinically, VEEV is indistinguishable from dengue and other arboviral diseases, often humans infected with VEEV symptoms appear within 2-5 days, and range from febrile, or flu-like symptoms, such as malaise, fever, chills, and myalgia, to coma and death are registered in ~1% of cases [3]. The confirmatory diagnosis requires specialized laboratory tests that are difficult to afford in regions with limited economic incomes.

Therefore, an endemic disease in developing countries remains largely unknown, and surveillance suggests that VEEV may represent up to 10% of the dengue burden in neotropical cities, or tens of thousands of cases per year throughout the Latin America [3,16].

The objective of this study was to detect antigens of the VEEV in tissues of bats.

## Materials and Methods

### Ethical approval

The ethics committee of the Faculty of Veterinary Medicine of the University of Córdoba, Colombia, approved the study. The committee took into account the rules of the National Environmental Authority of Colombia for researching with animal with non-commercial purposes. The permit for scientific research in biological diversity was also obtained, involving activities of collection, capture, hunting, fishing, and biological resource manipulation at the University of Córdoba through resolution 00914 of August 4, 2017. To avoid animal suffering, bats were initially premedicated with atropine (0.005 mg/kg; Laboratories ZOO, Bogota, Colombia) and acepromazine (0.11 mg/Kg; Laboratories ZOO, Bogota, Colombia) by intramuscularly route of administration and euthanized by overdose de 0.2 ml of sodium pentobarbital by intracardiac (Invet, Bogota, Colombia). Brain, heart, lung, liver, kidney, and spleen samples were extracted in the field. The capture site dissections were performed with the use of biosecurity equipment and materials necessary for this type of study [17,18]. Specimens in danger of extinction and pregnant or lactating females bats were released.

### Study period, type of study, sample size, and geographical areas

Between 2015 and 2017, a prospective descriptive cross-sectional study was carried out. A non-probabilistic sampling was carried out, and 286 bats were captured. The geographical areas chosen were two Córdoba and Sucre departments of the Colombian Caribbean area. In total, there were 12 capture sites, eight in the department of Córdoba, and four in the department of Sucre.

### Capture of bats

The fog nets (6 m×2 m) were placed in places near water sources, forests, wetlands, tree plantations, farmlands and pastures, livestock pens, and sites near rural residences. Between 18:00-4:00 h, the nets were placed, and they were reviewed every 15 min to collect the specimens, the taxonomic identification was carried out by standard morphometric data such as total length, tail length, leg, and length of the forearm [19]. Biological data of bats were collected, such as sex, reproductive status, relative age, weight, and presence of ectoparasites. The data were geo-referenced and a descriptive statistic for each of the qualitative and quantitative variables was entered into an Excel database.

### Molecular methods

RNA extraction was performed with Trizol (Invitrogen™) following the manufacturer’s protocol. The cDNA synthesis was obtained with the reverse transcriptase (RT) enzyme Moloney Murine Leukemia Virus (M-MLV) (Invitrogen™) using random primers. The reverse transcription reaction was performed in a single cycle at 42°C using the M-MLV RT. A fragment of the nsP4 gene encoding the polymerase of the alphaviruses was amplified by RT-polymerase chain reaction (PCR)-nested, using the primers Alpha1+, Alpha1− and Alpha2+, Alpha2− (Invitrogen™) proposed by Sánchez-Seco *et al*. [20]. As control of species and internal control, complementary primers were used to a sequence of a mitochondrial gene mtDNA from bats [21], as a positive control of the lyophilized vaccine prepared with the attenuated virus of equine encephalomyelitis TC83 was used; molecular water grade as a negative control was used. The positive samples were reamplified, and the amplicons were sequenced using the Sanger method [22]. For the analysis of pathological anatomy and immunohistochemistry, positive and negative samples were included using molecular biology.

### Pathological anatomy analysis

Samples of brain, heart, lung, spleen, liver, and kidney tissues, positive by RT-PCR, and confirmed by Sanger sequencing, were analyzed by histopathology and immunohistochemistry. The samples were processed by the conventional histological method, which consists of dehydration in scaling concentration alcohols and their inclusion in paraffin. Sections with 4 μm thickness were stained with hematoxylin/eosin and processed for optical microscopy studies.

### Immunohistochemistry

For the detection of VEEV, a Mouse Encephalitis, Equine, Venezuelan Monoclonal Antibody (Chemicon International, Inc. USA) was performed. Histological specimens of 4 μm were placed on positively charged slides, and these were placed at 60°C for 2 h. The antigenic recovery was made under pressure (Cuisinart Pressure Cooker Model CPC-600). For antigenic recovery, the heat-induced epitope recovery technique was used using the Trilogy^®^ reagent (Cell Marque, Rocklin, CA, USA) at a 1:100 dilution for 15 min at 125°C) that allowed dewaxing, rehydration, and antigenic recovery simultaneously. The slides were washed 3 times in phosphate-buffered saline (PBS), then introduced in 9% H_2_O_2_ diluted in methanol to block the peroxidase, washed with PBS, the cuts were delineated with Dako Pen. The tissues were covered with the monoclonal antibody at a dilution of 1:100, and the HiDefTM amplifier was applied and incubated for 10 min at room temperature. Later three washes with PBS were made, and the HiDefTM horseradish peroxidase polymer detector was applied and washed with PBS. The tissue was covered with the Dako Liquid DAB+Substrate Chromogen System, washed in PBS, and contrast stained with hematoxylin for 1 min, the sections were dehydrated with alcohols of increasing concentration. Finally, they were immersed in xylol and covered with coverslips using Entellan^®^. In all cases, as a negative control of the technique, the primary antibody was replaced by 1% bovine serum albumin in PBS. Mouse brain slices inoculated with VEEV were used as positive controls [23]. Bat tissues RT-PCR negative were used as negative controls.

## Results

Twenty-three species belong to six families were grouped; Table-1 shows the distribution of capture bats.

**Table-1:** Distribution of bat species by food sources.

Food source	Captured species	No.	Food source	Captured species	No.
Insectivorous	*Molossus molossus*	14	Frugivorous	*Artibeus planirostris*	99
	*Uroderma bilobatum*	11		*Carollia perspicillata*	38
	*Saccopteryx bilineata*	4		*Artibeus lituratus*	30
	*Eptesicus brasiliensis*	1		*Sturnira lilium*	20
	*Rhogeessa yo*	2		*Carollia brevicauda*	1
	*Eumops glaucinus*	1		*Carollia castanea*	1
	*Lasiurus ega*	1	Piscivorous	*Noctilio albiventris*	3
	*Micronycteris microtis*	1		*Noctilio leporinus*	3
	*Myotis nigricans*	1	Opportunistic piscivorous	*Phyllostomus discolor*	42
	*Saccopteryx leptur*	1		*Trachops cirrhosus*	1
	*Molossops temminckii*	1			
Nectarivorous	*Glossophaga soricina*	6	Sanguinovorous	*Desmodus rotundus*	4

**Figure-1:**
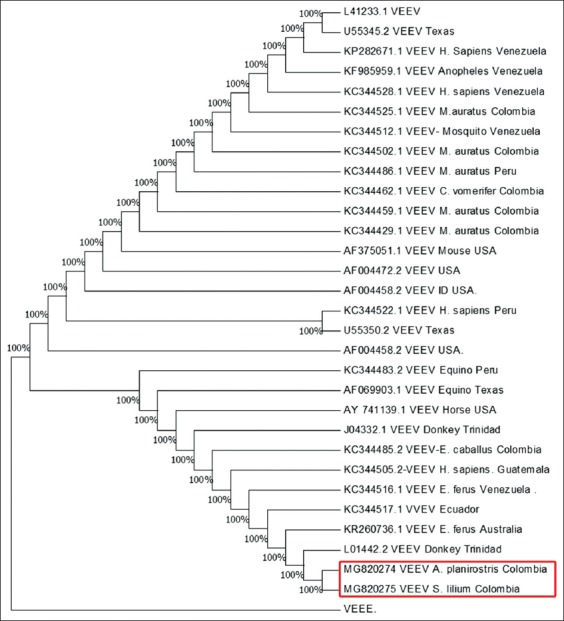
Phylogeny of the Venezuelan equine encephalitis virus detected in frugivorous bats.

The phylogenetic analysis demonstrates that sequences were closely related to the VEEV and only showed 3% of sequence divergence with previous sequences registered in GenBank (Figure-1).

Figure-1 Shows phylogeny of the VEEV obtained withMEGA Version 7.0 (https://www.megasoftware.net/) detected in frugivorous bats, and compared with other alphaviruses reported in Genbank. The consensus sequence obtained in this study presented a similarity of 97%, and 100% coverage with sequences of the gene was encoding the nsp4 protein registered in GenBank: KC344505.2 Guatemala/human [24] KC344485.2 (strain CoAn5384 equine isolated from Cali, Colombia) [25]. That is, the sequences found in frugivorous bats of the Colombian Caribbean are similar to sequences of the epizootic cycle of VEEV (Subtype I, varieties AB and C), which are the main responsible for outbreaks in humans and equines.

### Pathological anatomy analysis

**Figure-2:**
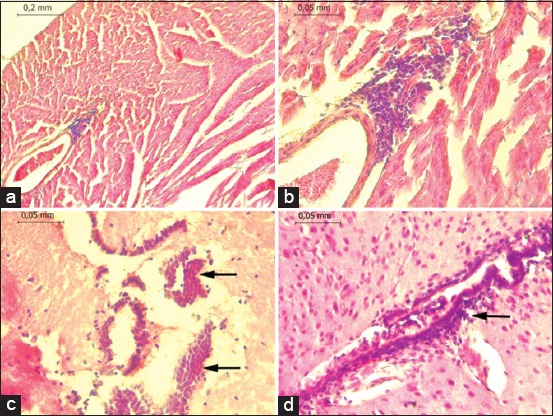
Pathological lesions in samples of positive specimens detected by Reverse transcription-polymerase chain reaction. (a and b) Non-suppurative myocarditis staining with hematoxylin-eosin (100×). Perivascular edema, diffuse gliosis, satellitosis, neuronal death, demyelination. (c) Lymphocytic meningitis, non-suppurative viral type meningitis (d).

VEEV antigens and pathological lesions were detected in two specimens, and both were also positive by RT-PCR. Figure-2 shows the findings of the pathological anatomy found in the tissues of *Sturnira lilium* in the central nervous system, severe meningitis, and non-suppurative vasculitis of the lymphocytic type, perivascular and perineuronal edema, diffuse gliosis, satellitosis, neuronophagia, neuronal death, and neuropil vacuolization. In the liver, there is a severe mononuclear infiltrate of lymphocytic type in the portal triad and multifocally in the periacinar and centrolubulillar area and vascular changes in hepatocytes. The final diagnosis was a severe non-suppurative meningoencephalitis of viral type and severe non-suppurative hepatitis of infectious viral type. Concerning the other specimen *Artibeus planirostris*, in the lung, there was a hyperplasia of a lymphoid type associated with bronchi, thickening of alveolar septa, mononuclear lymphocytic alveolitis, and mixed infiltrate with mononuclear predominance. In the liver, mild non-suppurative hepatitis of the lymphocytic type was observed.

### Immunohistochemical analysis

**Figure-3:**
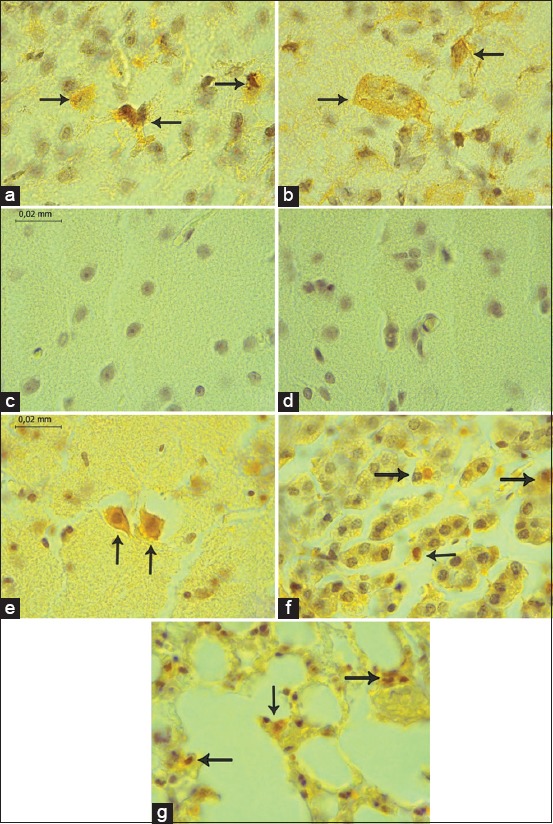
Controls and distribution of Venezuelan equine encephalitis virus (VEEV) antigens. (a and b) Positive brain controls in 3-day-old mice inoculated with the VEEV virus with immunostaining. (c and d) Negative controls. Histological section of the brain with the absence of immunostaining for VEEV. Distribution of VEEV antigens. Immunoreaction is observed in neurons (arrow) (e); splenic tissue (f) (arrow); and (g) septum cells.

Tissues of brain, spleen, and lung of frugivorous bats that were previously positive by RT-PCR and sequenced by the Sanger method were tested with specific antibodies for the VEEV. In Figure-3: (a and b), positive controls (c and d) negative controls, (e, f, and g) positive samples. It is possible to observe VEEV antigens in the brain, spleen, and lung.

## Discussion

The role of bats in the cycle of transmission of VEEV has not yet been established; in this study, for the first time in the world, evidence of the presence of VEEV-specific antigens in frugivorous bats’ tissues is shown. A fragment of the *Alphavirus* nsP4 protein whose sequences were similar to that of VEEV was amplified. The sequence obtained from the sample registered with an access code to GenBank: MG820274 showed a 97.7% similarity with the sequence of the gene that codes for the precursor of the structural and non-structural protein of strain VEEV registered in GenBank with accession number KR260736.1 (strain COAN5506 equine isolate from Colombia). The obtained sequence had coverage of 100% and an E-value=1.03e-55, position 1 of the sequence is homologous to position 6335 of the sequence KR260736.1, and nucleotide 131 is homologous to position 6465.

The sequence registered with an access code to GenBank: MG820274

TTGTAATGTGACGCAAATGAGAGAATT GCCCGTATTGGATTCGGCGGCCTTTAATGTG GAATGCTTCAAGAAATATGCGTGTAATAAT GAATATTGGGAAACGTTTAAAGAAAACCCCA TCAGGCTTACTGAAGAAAACGTGGTAAATTA CATTACCAAATTAAAAGGCCCCAAAGCCGCC GCCTTCTT1

GTAAGCCTGATGGGGTTTTCTTT AAACGTTTCCCAATATTCATTATTAC ACGCATATTTC 60

GTAAGCCTGATGGGGTTTTCTTTAAACG TTTCCCAATATTCATTATTACACGCATATTTC

Code GenBank: KR260736.1

6335GTAAGCCTGATGGGGTTTTCTTT AAACGTTTCCCAATATTCATTAT TACACGCATATTTC 6394

61TTGAAGCATTCCACATTAAAGGCCGCC GAATCCAATACGGGCAATTCTCTCATCTGCGT C120

TTGAAGCATTCCACATTAAAGGCC GCCGAATCCAATACGGGCAATTCTCTCAT TGCGT

Code GenBank: KR260736.1

6395TTGAAGCATTCCACATTAAAG GCCGCCGAATCCAATACGGGCAAT TCTCTCATTTGCGTG 6454

Code GenBank MG820274

121 ACATTACAATT

ACATT CAATT 131

Code GenBank KR260736.1

6455 ACATTGCAATT 6465

In this study, the nsp4 non-structural protein was amplified because it is the most conserved protein in alphaviruses and encodes RNA-dependent RNA polymerase. This enzyme is responsible for replicating viral RNA. The choice of this region was due to its high degree of conservation in all alphaviruses. With this chosen, the risk of losing positive samples with possible mutations of the virus in the junction zone of the primers was reduced. On the other hand, it is an area in which numerous previous studies of phylogeny have been carried out and for which the sequences of all the alphaviruses previously described are known. Although no experimental trials were conducted in this study to determine the biological function of the detected mutations, they may likely cause clinical complications in patients; therefore, this type of study would be essential to understand the biological and phylogenetic characteristics of this virus.

The emergence of zoonotic diseases is increasing worldwide, and mammals, including bats, are significant sources of emerging and re-emerging pathogens. Bats are natural reservoirs of a variety of emerging viruses that cause important diseases in humans and pets. It is recognized that bats of certain species are capable of transmitting the rabies virus, but recent observations of outbreaks and epidemics of human diseases caused by bat-transmitted viruses have once again drawn attention to these mammals. Bats harbor more zoonotic viruses per species than rodents and are now recognized as an essential source of zoonotic agents [26-29].

Human pathogens viruses such hepacivirus, pegivirus [30], *Influenza A* [31], *Hanta* [32], paramyxovirus, respiratory syncytial virus [33], arenaviruses [34], Hendra virus, Nipah virus, Ebola virus, Marburg virus, and coronaviruses have also found in bats [26-29].

Some seroprevalence studies in countries of the Americas have reported that bats can be infected naturally by the VEEV [12-15], and they are likely to act as reservoirs to feed mosquitoes that feed on blood, and then they can transmit the virus to domestic animals and humans. Although in the bats captured in this investigation, no apparent neurological symptoms were observed that were related to VEEV, a diffuse neutrophilic infiltrate was found in the brain with neuronal necrosis and multifocal vasculitis detected by histopathological diagnosis compatible with non-suppurative encephalitis of viral type, these presumptive lesions of encephalitis were confirmed by specific labeling of VEEV antigens.

The results of pathological anatomy showed that the histological characteristics of the lesions found were concordant with the detection of VEEV viral antigens in tissues of frugivorous bats naturally infected with the VEEV. These histological lesions were not observed in the tissues of bats included in this study and were negative by molecular tests and immunohistochemistry to VEEV.

Several arboviruses that are maintained in more than one host/vector species are considered generalists, such as St. Louis EEV, West Nile virus, Japanese encephalitis virus, EEV, western EEV (WEEV), and VEEV.

Immunohistochemical detection of VEEV antigens in paraffin-embedded tissues provides a rapid and reliable means of confirmation of histological diagnosis. The results of this study correlate well with the results of molecular virus detection and sequencing. These results indicate that the IHC technique constitutes one of the useful tools of specific diagnosis due to its high sensitivity and specificity, the reason why it can also be used for the identification of other tropical etiologies. Unfortunately, this technique has a high cost to be transferred to regional reference laboratories in Colombia.

The analysis of the dynamic maintenance of these viruses appears to link to a transmission network instead of a transmission cycle [11]. The mechanisms for the maintenance of arboviruses are intrinsically complicated and must be taken into account and incorporated when designing and constructing mathematical models that allow predicting their enzootic/epidemic activity in a given ecosystem (wild, urban, and agricultural, as well). Consequently, to better understand the activity pattern and the transmission networks of arboviruses, it is essential to understand the assembly of host species and vectors, their interactions, and fluctuations through time and space. Although the natural infection rate found in this study by molecular methods is low (0.2%), it poses the challenge of being able to complement surveillance with serological detection to establish the presence of the virus in regions where the disease is absent in humans. The role of naturally infected frugivorous bats is challenging due to the short period during which these infected animals “could be viremic,” and the serological detection of specific immunoglobulin offers the most effective means of confirming it.

On the other hand, the “viremic” animals in their wild cycle could reach the water sources and have contact with the mosquitoes, and these infect domestic animals that are in contact with humans. Likewise, the relative proximity of frugivorous bats and humans due to disruption of their natural habitat makes it possible for the viral transmission to occur through the consumption of contaminated fruits. Therefore, the approach of these arboviruses requires designs that contemplate an integral and connectable perspective in a network that allows to improve the understanding of the dynamics of the viruses and thus improve the preventive measures in vector control and public health policies.

Finally, this study highlights the circulation of VEEV in frugivorous bats captured in rural areas of the departments of Córdoba and Sucre, Colombia. The finding is useful for public health authorities since they can establish permanent surveillance of wild VEEV cycles that anticipate a possible outbreak to implement prevention and control measures.

## Conclusion

The presence of VEEV antigen was confirmed in the brain, lung, and spleen of frugivorous bats that were also positive by RT-PCR, sequencing, and histopathology.

## Authors’ Contributions

CG: Designed the study, writing of the manuscript and analyzed the data. AC: Helped in the practical experiment, drafted the manuscript and analyzed the data. TO: Helped in the practical experiment, drafted the manuscript and analyzed the data. SM: Helped in design of the study, supervised and drafted the manuscript. JC: Helped in the practical experiment, drafted the manuscript and analyzed the data. VR: Helped in the practical experiment, drafted the manuscript and analyzed the data. LTMF: Helped in the practical experiment, drafted the manuscript and analyzed the data. All authors read and approved the final manuscript.
